# Proximal median nerve neuropathy: electrodiagnostic and ultrasound findings in 62 patients

**DOI:** 10.3389/fneur.2024.1468813

**Published:** 2024-12-05

**Authors:** Lisa B. E. Shields, Vasudeva G. Iyer, Yi Ping Zhang, Christopher B. Shields

**Affiliations:** ^1^Norton Neuroscience Institute, Norton Healthcare, Louisville, KY, United States; ^2^Neurodiagnostic Center of Louisville, Louisville, KY, United States

**Keywords:** neurology, proximal median nerve neuropathy, iatrogenic nerve injury, electrodiagnostic studies, ultrasound study of nerve

## Abstract

**Objectives:**

Proximal median nerve (PMN) neuropathies are caused by lesions proximal to the carpal tunnel, which include the forearm, elbow, upper arm, and brachial plexus. Differentiating between carpal tunnel syndrome and PMN neuropathies is important to guide management and is based on clinical, electrodiagnostic (EDX), and ultrasound (US) findings. This study describes the clinical, EDX, and US features in 62 patients with PMNs.

**Methods:**

All patients underwent EDX studies, and 52 (83.9%) had a US study. The patients were assigned to one of the following four localization zones of PMN neuropathies based on clinical and EDX criteria: Zone 1: extends from the fascicles in the brachial plexus contributing to the median nerve to the innervation of the pronator teres (PT); Zone 2: distal to the branch to the PT and proximal to the origin of the anterior interosseous nerve (AIN); Zone 3: involves the origin of the AIN; and Zone 4: distal to the origin of the AIN and proximal to the carpal tunnel. The localization was based on the pattern of muscle weakness, topography of EMG abnormalities, and US study findings.

**Results:**

The anatomical locations of the PMN neuropathies based on clinical, EDX, and US findings were as follows: Zone 1 in 38 patients (61.3%), Zone 2 in 6 patients (9.7%), Zone 3 in 7 patients (11.3%), and Zone 4 in 11 patients (17.7%). The most common etiology among all 62 patients was iatrogenic injury (30 [48.4%]), followed by non-iatrogenic trauma (20 [32.2%]). The following EDX findings were noted: prolonged distal motor latency (29 [46.8%]), decreased motor nerve conduction velocity in the forearm (22 [35.5%]), low amplitude or absent compound muscle action potentials (50 [80.6%]), and abnormal or absent sensory nerve action potentials (50 [80.6%]). Of the 52 (83.9%) patients who underwent US studies, a total of 22 (42.3%) patients showed an increased cross-sectional area of the median nerve. A neuroma was observed in 9 patients (17.4%).

**Conclusion:**

It is often possible to localize the site of the median nerve involvement and gain insight into the underlying cause based on clinical and EMG findings, but in certain cases, a US study may be necessary to confirm the location.

## 1 Introduction

The median nerve arises from the medial and lateral cords of the brachial plexus and has contributions from C5-T1 nerve roots (1–8). It descends lateral to the brachial artery and between the biceps brachii and brachialis muscles. The median nerve then traverses the antecubital fossa and runs deep to the bicipital aponeurosis and anterior to the brachialis muscle. It then passes between the superficial and deep heads of the pronator teres (PT). In the forearm, the median nerve travels between the flexor digitorum superficialis and profundus muscle bellies. The most common median nerve neuropathy is carpal tunnel syndrome (CTS), where the median nerve is compressed under the flexor retinaculum (4–6). Proximal median nerve (PMN) neuropathies denote lesions of the median nerve at locations proximal to the carpal tunnel, which include the forearm, elbow, upper arm, and brachial plexus. They are often underdiagnosed or misdiagnosed as CTS due to the similarity of symptoms and overlapping pathologies, which may co-exist in the same patient (4, 9).

Several proximal median nerve entrapment sites have been identified (from proximal to distal): (1) beneath the ligament of Struthers, which extends from the supracondylar process of the distal humerus; (2) bicipital aponeurosis (lacertus fibrosus); (3) proximal insertion of the PT humeral head; (4) passage between the two heads of the PT; (5) dysplasia of the deep PT head; (6) common arcade between the PT and flexor digitorum superficialis (FDS); (7) fibrous arch/arcade of the FDS; and (8) Gantzer muscle (1, 5, 7, 9–14). At several entrapment sites, mechanical compression causing a compromised arterial supply to the median nerve may lead to PMN neuropathies (10). At the level of the axilla, the nerve is vulnerable to compression in the medial fascial compartment (15).

Both PT and anterior interosseous nerve (AIN) syndromes involve compression/entrapment of the median nerve proximal to the carpal tunnel (4, 7, 14, 16). In 1951, Seyffarth described a case of median nerve compression in the proximal forearm and coined the term “pronator syndrome” (17). PT syndrome is considered to be a compressive/entrapment neuropathy of the median nerve in the proximal forearm with symptoms and signs that are similar to those of CTS (1, 10, 11, 18–20). AIN syndrome is a pure motor palsy resulting from compression/entrapment of the AIN close to its origin, most often by the deep and superficial heads of the PT or fibrous arcade of the FDS (10, 16).

The median nerve may be injured by either iatrogenic (excessive traction or use of retractors during surgery) or non-iatrogenic causes (direct penetrating trauma by a gunshot wound, knife wound, or broken glass) (3). EDX and US studies are invaluable in identifying the lesion site and distinguishing between the different underlying pathologies (16). These studies, especially US, are valuable to ascertain the etiology of the PMN neuropathies, which can potentially direct medical or surgical management (5).

A large series of patients with PMN neuropathies that includes EDX and US data is lacking in the literature. In this report, we describe 62 patients with PMN neuropathies, confirmed by EDX studies, 52 (83.9%) of whom also had US studies. The clinical, EDX, and US findings are presented. The etiologies and mechanisms of PMN neuropathies based on the specific anatomical zone of involvement are discussed. The significance of US in complementing the EDX studies for localization and determining the underlying cause is also highlighted.

## 2 Materials and methods

### 2.1 Electrodiagnostic studies and ultrasound studies

**Figure 1:**
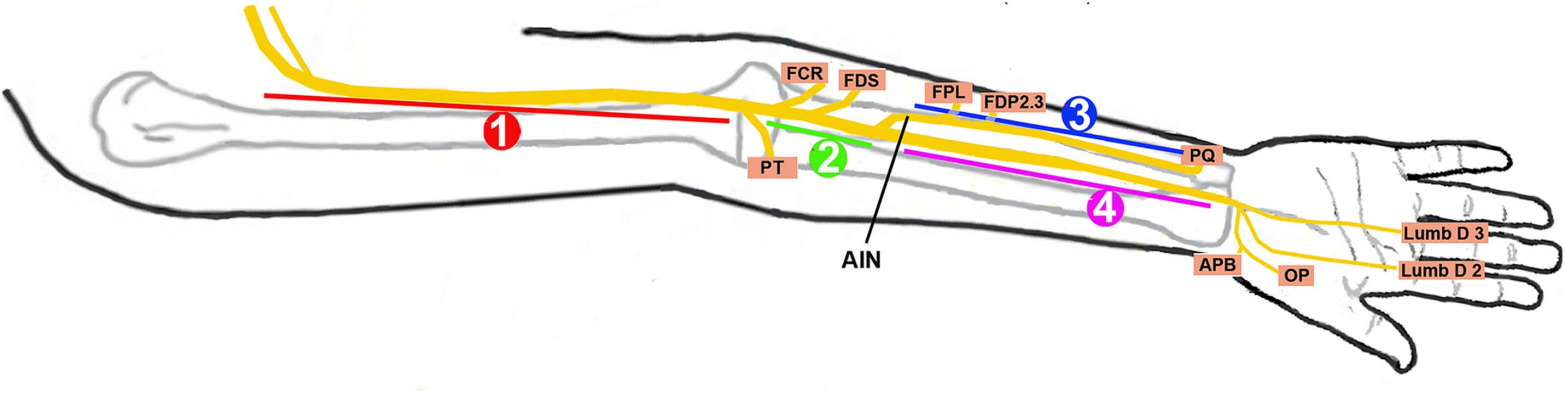
Localization zones of proximal median neuropathy. Zone 1, at or proximal to the branch to the pronator teres; Zone 2, distal to the branch to the pronator teres and proximal to the origin of the anterior interosseous nerve (AIN); Zone 3, involving the AIN; Zone 4, distal to the origin of the AIN and proximal to the carpal tunnel; FCR, flexor carpi radialis; FDS, flexor digitorum superficialis; FPL, flexor pollicis longus; FDP 2 and 3, flexor digitorum profundus to the second and third digits; PQ, pronator quadratus; APB, abductor pollicis brevis; OP, opponens pollicis; Lumb D 2 and 3, lumbricals of the second and third digits.

Under an Institutional Review Board (IRB)-approved protocol, we performed a 14½-year (October 2010–June 2024) retrospective analysis of patients referred to our neurodiagnostic center for EDX studies to evaluate PMN neuropathies. In each patient, PMN neuropathy was assigned a specific anatomic zone based on the localization of median nerve involvement by EMG and US findings (Figure 1; Table 1). The patients underwent a focused neurological examination of the upper extremities, followed by nerve conduction velocity (NCV) and needle EMG studies. The EDX studies were performed in our American Association of Neuromuscular & Electrodiagnostic Medicine (AANEM)-accredited facility using the standard protocol of our laboratory (21).

**Table 1:** Localization by zones in median nerve neuropathy.

Zone	Location	Muscles showing denervation	Potential causes
1	At or proximal to the innervation of the PT	All muscles including the PT, FPL, and APB	Injuries/compression at the axilla, upper arm, and entrapment at the ligament of Struthers
2	Distal to branch to PT and proximal to the origin of the AIN	All muscles except PT	Injuries/compression/entrapment at the PT, lacertus fibrosus, and FDS arch
3	AIN	FPL and FDP to digit 2	Injuries/compression/entrapment and fascicular involvement as in PaTS
4	Distal to origin of AIN	APB	Injuries/compression
5	At the carpal tunnel	APB	Entrapment at the carpal tunnel

PT, pronator teres; FPL, flexor pollicis longus; APB, abductor pollicis brevis; AIN, anterior interosseous nerve; FDS, flexor digitorum superficialis; FDP, flexor digitorum profundus; PaTS, Parsonage–Turner syndrome.

The standard protocol in our lab is to stimulate the median nerve at the wrist and the elbow with the recording electrodes over the abductor pollicis brevis (APB) (second lumbrical, if no response is seen over the APB). Additional studies were performed when indicated: (1) if the clinical findings suggested Zone 1 and intact CMAP were recorded over the APB, stimulation was extended to the upper arm and axilla to detect the site of conduction block in the proximal portions of the median nerve and (2) if the findings suggested the AIN as the site of involvement, the recording electrodes were placed over the pronator quadratus (PQ) to calculate the motor conduction velocity in the AIN (22, 23). A comparison study was performed on the unaffected side.

Needle EMG was performed in a “topographic mapping sequence,” starting at the APB, followed by flexor pollicis longus (FPL) and PT. Based on the initial findings, additional muscles such as the flexor carpi radialis (FCR), FDS, flexor digitorum profundus (FDP), and PQ were studied to get more precise localization and to identify selective fascicular involvement—common in Parsonage-Turner syndrome (PaTS). A proximal inflammatory (or even partial trauma) lesion can potentially cause distal slowing of motor conduction velocity due to anterograde demyelination and loss of fast conduction axons; this can also be caused by slow conduction in regenerating axons.

A US study was conducted using a GE Logiq E system with a 5–16 and/or 12–18 MHz probe, tracking the median nerve. The cross-sectional area (CSA) of the nerve was measured along the course of the median nerve at the wrist, proximal forearm, elbow, and upper arm.

### 2.2 Categorization of inflammatory AIN lesions

While PaTS is considered an immune-mediated acute brachial plexus neuropathy, the majority of patients present with symptoms and signs of single nerve involvement, most often anterior or posterior interosseous, axillary, or suprascapular nerves. MR neurography has shown changes in the brachial plexus but more often hourglass appearance in fascicles, which finally become one of the above-mentioned nerves (24). In our study, we classified PMN neuropathies into zones based on clinical/EMG patterns. If the pattern suggested location to the AIN in patients with a clinical picture of PaTS, they were included in Zone 3. Patients with a clinical diagnosis of PaTS but showed changes in the PT were included in Zone 1. Patients clinically diagnosed with PaTS (acute onset of scapular pain followed by muscle weakness in the distribution of one or more nerves derived from the brachial plexus) may have features of Zone 1, 2, or 3.

The diagnosis of PaTS is based on the typical clinical picture, specifically the acute onset of pain in the scapular area, followed by the onset of muscle weakness in the distribution of usually a single nerve derived from the brachial plexus. By the time the patients were referred to our lab, it was too late to perform MR neurography of the brachial plexus.

### 2.3 Inclusion and exclusion criteria

The inclusion criteria were (1) patients with clinical features of PMN neuropathies (Zones 1–4) and (2) EDX findings confirming PMN neuropathies. Patients with evidence of distal median neuropathy at the carpal tunnel (Zone 5) were excluded from the study. Several metrics were collected including age, sex, symptom laterality (right/left), hand dominance (right/left/ambidextrous), clinical history, specific zone of PMN neuropathies, etiology (trauma [iatrogenic vs. non-iatrogenic] or other causes), symptom onset (acute versus gradual), EDX findings, and US features. Specific NCV data that were compiled included distal motor latency, motor nerve conduction velocity (MNCV) in the forearm, amplitude of the compound muscle action potentials (CMAPs), and latency and amplitude of sensory nerve action potentials (SNAPs). Needle EMG was recorded from the APB, FPL, and PT muscles. US findings collected include CSA of the median nerve at different locations and altered echogenicity.

### 2.4 Institutional review board approval of research

Informed consent was obtained from all patients. The IRB determined that our study was exempt according to 45 CFR 46.101(b) under Category 4. The IRB number is 22.1060.

## 3 Results

### 3.1 Demographics and specific proximal median nerve localization

**Figure 2:**
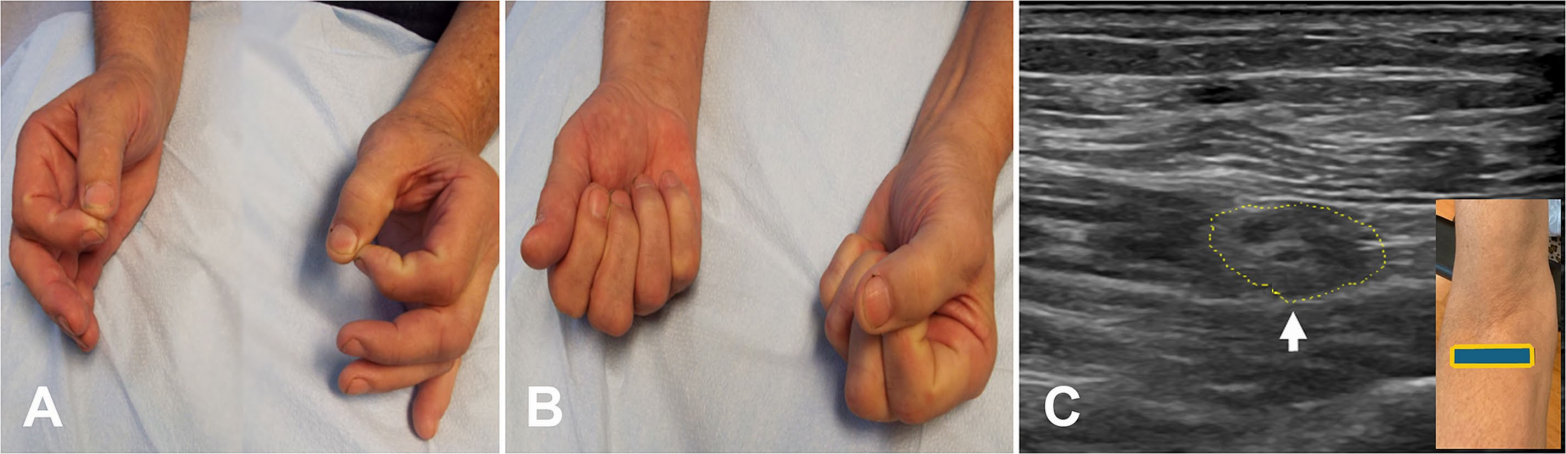
Acute onset of pronator teres syndrome. **(A)** Positive O sign on the right. **(B)** Weakness of the flexor pollicis longus on the right. **(C)** Ultrasound study showing enlarged median nerve (encircled by yellow dots, arrow) with a few large fascicles.

A total of 62 patients were diagnosed with PMN neuropathies based on EDX and US studies (Tables 2–7). The mean age was 56.4 years (range: 14–82 years), and the majority (36 [58.1%]) of the patients were male. The occurrence of PMN neuropathy was more common on the right side (32 [51.6%]). Fifty-four patients (87.1%) were right-hand dominant, 5 (8.1%) were left-hand dominant, and 3 (4.8%) were ambidextrous. The symptomatic side corresponded to hand dominance in 34 patients (54.8%). The PMN neuropathy localization was as follows: Zone 1 (38 patients [61.3%]), Zone 2 (6 patients [9.7%]), Zone 3 (7 patients [11.3%]), and Zone 4 (11 patients [17.7%]). Fifty-nine (95.2%) patients had an acute onset of symptoms, while 3 (4.8%) experienced gradual onset (Figures 2A–C).

**Table 2:** Demographics and etiologies of proximal median neuropathy.

Zone	Age (years) (mean)	Sex (male/female)	Side (left/right)	Etiology	Number of patients with specific etiologies
Total (*n* = 62)	56.4 (14–82)	M: 36 (58.1%) F: 26 (41.9%)	L: 30 (48.4%) R: 32 (51.6%)	Trauma: iatrogenic Trauma: non-iatrogenic Other	30 (48.4%) 20 (32.2%) 12 (19.4%)
Zone 1 (*n* = 38)	58.9 (14–82)	M: 19 (50.0%) F: 19 (50.0%)	L: 22 (57.9%) R: 16 (42.1%)	Trauma: iatrogenic Shoulder surgery Brachial artery puncture Dialysis port PICC line IV extravasation Axillary artery puncture Elbow joint repair Phlebotomy Restraint in hospital Trauma: non-iatrogenic Fracture of humerus Stab injury Glass cut injury Needle injury (drug user) Other PaTS Schwannoma	25 (65.8%) 7 (28.0%) 5 (20.0%) 4 (16.0%) 3 (12.0%) 2 (8.0%) 1 (4.0%) 1 (4.0%) 1 (4.0%) 1 (4.0%) 9 (23.7%) 6 (66.7%) 1 (11.1%) 1 (11.1%) 1 (11.1%) 4 (10.5%) 3 (75.0%) 1 (25.0%)
Zone 2 (*n* = 6)	52.3 (23–81)	M: 4 (66.7%) F: 2 (33.3%)	L: 2 (33.3%) R: 4 (66.7%)	Trauma: iatrogenic Trauma: non-iatrogenic Fracture radius Other Exertion	1 (16.7%) 2 (33.3%) 2 (100%) 3 (50%) 3 (100%)
Zone 3 (*n* = 7)	57.8 (47–75)	M: 5 (71.4%) F: 2 (28.6%)	L: 4 (57.1%) R: 3 (42.9%)	Trauma: iatrogenic Biceps tendon repair PaTS Trauma: non-iatrogenic Other PaTS	2 (28.6%) 1 (50.0%) 1 (50.0%) 0 (0%) 5 (71.4%) 5 (100%)
Zone 4 (*n* = 11)	50.2 (21–72)	M: 8 (72.7%) F: 3 (27.2%)	L: 2 (18.2%) R: 9 (81.8%)	Trauma: iatrogenic Elbow repair Repair of fractured radius Trauma: non-iatrogenic Laceration injury Penetrating injury Crush injury Glass cut injury Neuroma from a gunshot injury Other	2 (18.2%) 1 (50.0%) 1 (50.0%) 9 (81.8%) 3 (33.3%) 3 (33.3%) 1 (11.1%) 1 (11.1%) 1 (11.1%) 0 (0%)

Left, left; Right, right; PaTS, Parsonage–Turner syndrome.

**Table 3:** NCV, needle EMG, and ultrasound findings in proximal median neuropathy.

Modality	Description	Number of patients				
		Total	Zone 1 (*n* = 38)	Zone 2 (*n* = 6)	Zone 3 (*n* = 7)	Zone 4 (*n* = 11)
Abnormal NCV study (*n* = 62)	Prolonged distal motor latency	29 (46.8%)	17 (44.7%)	2 (33.3%)	4 (57.1%)	6 (54.5%)
	MNCV decreased in forearm	22 (35.5%)	16 (42.1%)	3 (50.0%)	0 (0%)	3 (27.3%)
	Low amplitude/no CMAP	50 (80.6%)	33 (86.8%)	5 (83.3%)	2 (28.5%)	10 (90.9%)
	Abnormal/no SNAP	50 (80.6%)	36 (94.7%)	3 (50.0%)	0 (0%)	11 (100%)
Abnormal needle EMG (*n* = 62)	APB	53 (85.2%)	37 (97.4%)	6 (100%)	0 (0%)	11 (100%)
	FPL	50 (82.0%)	38 (100%)	6 (100%)	7 (100%)	0 (0%)
	PT	38 (62.3%)	38 (100%)	0 (0%)	0 (0%)	0 (0%)
Ultrasound (*n* = 52)	Increased CSA median nerve	22 (42.3%)	12 (40.0%)	4 (80.0%)	2 (33.3%)	4 (36.4%)
	Hyperechoic median nerve	16 (30.8%)	14 (46.7%)	2 (40%)	0 (0%)	0 (0%)
	Hypoechoic median nerve	6 (11.5%)	3 (10.0%)	0 (0%)	3 (50.0%)	0 (0%)
	Neuroma	9 (17.3%)	2 (6.7%)	0 (0%)	0 (0%)	7 (63.6%)
	Normal study	10 (19.2%)	8 (26.7%)	0 (0%)	2 (33.3%)	0 (0%)
	Not done	10 (16.1%)	8 (21.0%)	1 (16.7%)	1 (14.3%)	0 (0%)

NCV, nerve conduction velocity; EMG, electromyography; MNCV, motor nerve conduction velocity; CMAP, compound muscle action potentials; SNAP, sensory nerve action potentials; APB, abductor pollicis brevis; FPL, flexor pollicis longus; PT, pronator teres; CSA, cross-sectional area.

**Table 4:** Zone 1 in proximal median neuropathy (38 patients).

Age (years)/Sex (M/F)	Side (R/L)	Etiology	NCV	Needle EMG	Ultrasound
25/M	L	PaTS	1 and 2	2 and 3	Normal
23/M	L	Needle injury: (drug user) to the ACF	1, 3, and 4	1, 2, and 3	Hyperechoic nerve at the ACF with increased CSA
87/F	L	Iatrogenic injury: brachial artery puncture, and hematoma	3and 4	1, 2, and 3	Hypoechoic at distal upper arm with increased CSA
34/F	R	Non-iatrogenic injury: stab injury to the upper arm	3 and 4	1, 2, and 3	Hyperechoic nerve at the upper arm with 2–3 large fascicles
65/F	R	Non-iatrogenic injury: fracture of the humerus	3 and 4	1, 2, and 3	Hyperechoic nerve at the upper arm with 1–2 large fascicles
64/M	L	Iatrogenic injury: brachial artery puncture, pseudoaneurysm, and seroma	3 and 4	1, 2, and 3	Hyperechoic nerve at the upper arm with 3–4 large fascicles
14/M	R	Non-iatrogenic injury: fractured humerus, brachial artery thrombosis, and thrombectomy	3 and 4	1, 2, and 3	Large hypoechoic fascicles
76/M	R	Schwannoma to the upper arm	1, 2, 3, and 4	1, 2, and 3	Hypoechoic mass with features of Schwannoma
64/F	R	Iatrogenic injury: dialysis port	2 and 3	1, 2, and 3	Not done
82/M	L	Iatrogenic injury: shoulder surgery	1, 2, 3, and 4	1, 2, and 3	Normal distally
52/M	R	Iatrogenic injury: PICC line	1, 2, 3, and 4	1, 2, and 3	Hyperechoic with increased CSA and 2–3 large fascicles at the distal upper arm
42/M	L	Iatrogenic injury: dialysis port	3 and 4	1, 2, and 3	Hyperechoic with increased CSA and 1–2 large fascicles
56/F	R	Iatrogenic injury: IV extravasation ACF	1, 2, 3, and 4	1, 2, and 3	Hyperechoic nerve with increased CSA
69/F	R	PaTS	1, 2, 3, and 4	1, 2, and 3	Median nerve normal at the wrist/forearm
61/M	L	Iatrogenic injury: brachial artery puncture	1, 2, 3, and 4	1, 2, and 3	Hyperechoic nerve with increased CSA and large hypoechoic fascicles
46/M	R	Iatrogenic injury: phlebotomy ACF	1, 2, and 4	1, 2, and 3	Increased CSA ACF
46/M	L	Iatrogenic injury: dialysis port	1, 2, 3, and 4	1, 2, and 3	Neuroma in continuity
82/F	L	Iatrogenic injury: PICC line	3 and 4	1, 2, and 3	Pseudoaneurysm of the brachial artery
76/M	L	Iatrogenic injury: brachial artery puncture	3 and 4	1, 2, and 3	Hyperechoic nerve with increased CSA and large fascicles
34/M	R	Non-iatrogenic injury: glass cut injury in the upper arm	3 and 4	1, 2, and 3	Neuroma in continuity
59/F	R	Non-iatrogenic injury: fracture of the humerus	4	1, 2, and 3	Normal at the wrist and forearm
69/M	R	Iatrogenic injury: PICC line	1, 2, 3, and 4	1, 2, and 3	Hyperechoic nerve with increased CSA
60/M	L	Iatrogenic injury: shoulder surgery	1, 3, and 4	1, 2, and 3	Normal in the wrist, forearm
38/F	L	Iatrogenic injury: IV extravasation ACF	1, 2, 3, and 4	1, 2, and 3	Hyperechoic nerve with increased CSA
81/F	L	Injury: fractured humerus	1, 2, 3, and 4	1, 2, and 3	Normal at the wrist and forearm
53/M	R	Iatrogenic injury: restraint in hospital	3 and 4	1, 2, and 3	Not done
86/F	L	Iatrogenic injury: shoulder surgery	3 and 4	1, 2, and 3	Not done
72/F	L	Iatrogenic injury: axillary artery puncture	3 and 4	1, 2, and 3	Hyperechoic nerve with increased CSA and 2–3 large fascicles
75/F	R	Iatrogenic injury: shoulder surgery	3 and 4	1, 2, and 3	Not done
72/F	R	Iatrogenic injury: shoulder surgery	1, 2, 3, and 4	1, 2, and 3	Diffuse increased CSA and enlarged fascicles
62/M	L	Iatrogenic injury: brachial artery puncture	3 and 4	1, 2, and 3	Not done
39/F	L	Iatrogenic injury: shoulder surgery	3 and 4	1, 2, and 3	Not done
70/M	L	Non-iatrogenic injury: fracture of the humerus	3 and 4	1, 2, and 3	Enlarged hypoechoic nerve
59/F	L	Iatrogenic injury: dialysis port	3 and 4	1, 2, and 3	Not done
69/F	L	PaTS	4	1, 2, and 3	Normal at the wrist, forearm, and upper arm
55/F	L	Non-iatrogenic injury: fracture of the humerus	1, 2, and 4	1, 2, and 3	Hyperechoic with increased CSA
47/M	L	Iatrogenic injury: elbow joint repair	1, 2, 3, and 4	1, 2, and 3	Not done
73/F	R	Iatrogenic injury: shoulder surgery	3 and 4	1, 2, and 3	Normal at wrist, forearm, and upper arm

L, left; R, right; PaTS, Parsonage–Turner syndrome; FPL, flexor pollicis longus; PT, pronator teres; FCR, flexor carpi radialis; ACF, antecubital fossa; CSA, cross-sectional area; APB, abductor pollicis brevis; AV shunt, arteriovenous shunt; PICC line, peripherally-inserted central catheter line; MVA, motor vehicle accident; FDS, flexor digitorum superficialis; MNCV, motor nerve conduction velocity; CMAP, compound muscle action potentials; SNAP, sensory nerve action potentials. Nerve conduction: 1. Prolonged distal motor latency. 2. MNCV decreased in the forearm. 3. Low amplitude/no CMAP. 4. Abnormal/no SNAP. Needle EMG abnormality: 1. APB. 2. FPL. 3. PT.

**Table 5:** Zone 2 in proximal median neuropathy (six patients).

Age (years)/Gender (M/F)	Side (R/L)	Etiology	NCV	Needle EMG	Ultrasound
50/M	R	Undue exertion	1, 2, and 3	1 and 2	Enlarged, hyperechoic nerve proximal forearm, and large fascicles distally
66/M	R	Non-iatrogenic injury: fracture of radius	3 and 4	1 and 2	Not done
81/M	R	Undue exertion: lifted lawn mower	3 and 4	1 and 2	Increased CSA proximal forearm
23/M	L	Undue exertion: pressure washer	2, 3, and 4	1 and 2	Increased CSA and hyperechoic
63/F	L	Non-iatrogenic injury: fracture of radius	1, 2, and 3	1 and 2	Increased CSA
31/F	R	Iatrogenic: elbow surgery	1 and 2	1 and 2	Increased CSA and hypoechoic fascicles in the antecubital fossa

L, left; R, right; CSA, cross-sectional area; APB, abductor pollicis brevis; FPL, flexor pollicis longus; PT, pronator teres; MNCV, motor nerve conduction velocity; CMAP, compound muscle action potentials; SNAP, sensory nerve action potentials. Nerve conduction: 1. Prolonged distal motor latency. 2. MNCV decreased in the forearm. 3. Low amplitude/no CMAP. 4. Abnormal/no SNAP. Needle EMG abnormality: 1. APB. 2. FPL. 3. PT.

**Table 6:** Zone 3 in proximal median neuropathy (seven patients).

Age (years)/Sex (M/F)	Side (R/L)	Etiology	NCV	Needle EMG	Ultrasound
62/M	R	PaTS	1	2	Large fascicles median nerve at the elbow
50/M	R	PaTS		2	Small cyst in the vicinity of the AIN, probably an incidental finding
62/F	L	PaTS		2	Normal
75/F	L	PaTS	1, 3	2	Normal
51/M	R	Iatrogenic injury: biceps tendon repair	1, 3	2	Increased CSA of the median nerve in the proximal forearm
47/M	L	PaTS	1	2	Increased CSA of the median nerve in the proximal forearm and increased CSA of the AIN
58/M	L	PaTS		2	Not done

L, left; R, right; PaTS, Parsonage–Turner syndrome; AIN, anterior interosseous nerve; CSA, cross-sectional area; APB, abductor pollicis brevis; FPL, flexor pollicis longus; PT, pronator teres; MNCV, motor nerve conduction velocity; CMAP, compound muscle action potentials; SNAP, sensory nerve action potentials. Nerve conduction: 1. Prolonged distal motor latency. 2. MNCV decreased in the forearm. 3. Low amplitude/no CMAP. 4. Abnormal/no SNAP. Needle EMG abnormality: 1. APB. 2. FPL. 3. PT.

**Table 7:** Zone 4 in proximal median neuropathy (11 patients).

Age (years)/Sex (M/F)	Side (R/L)	Etiology	NCV	Needle EMG	Ultrasound
19/M	R	Iatrogenic injury: elbow repair with palmaris longus tendon harvest	1, 3, and 4	1	Increased CSA
22/M	L	Non-iatrogenic: penetrating injury forearm	3 and 4	1	Neuroma in continuity
72/M	R	Iatrogenic injury: fracture of the radius repair	1, 2, 3, and 4	1	Increased CSA, large fascicles
49/F	R	Non-iatrogenic: laceration injury to the distal forearm	1, 2, 3, and 4	1	Neuroma in continuity
21/F	L	Non-iatrogenic: laceration injury to the forearm	3 and 4	1	Neuroma in continuity
79/M	R	Non-iatrogenic: neuroma from gunshot injury to the forearm	3 and 4	1	Neuroma with fascicular discontinuity
57/F	R	Non-iatrogenic: glass-cut injury to the forearm	1, 3, and 4	1	Neuroma in continuity
64/M	R	Non-iatrogenic: crush injury to the forearm	1, 2, 3, and 4	1	Increased CSA
56/M	R	Non-iatrogenic: laceration injury to the forearm	3 and 4	1	Neuroma with fascicular discontinuity
68/M	R	Non-iatrogenic: penetrating injury to the forearm	1 and 4	1	Increased CSA
45/M	R	Non-iatrogenic: penetrating injury to the forearm	3 and 4	1	Neuroma with fascicular discontinuity

L, left; R, right; CSA, cross-sectional area; APB, abductor pollicis brevis; FPL, flexor pollicis longus; PT, pronator teres; MNCV, motor nerve conduction velocity; CMAP, compound muscle action potentials; SNAP, sensory nerve action potentials. Nerve conduction: 1. Prolonged distal motor latency. 2. MNCV decreased in the forearm. 3. Low amplitude/no CMAP. 4. Abnormal/no SNAP. Needle EMG abnormality: 1. APB. 2. FPL. 3. PT.

### 3.2 Etiologies by localization to specific zones of PMN neuropathy

**Figure 3:**
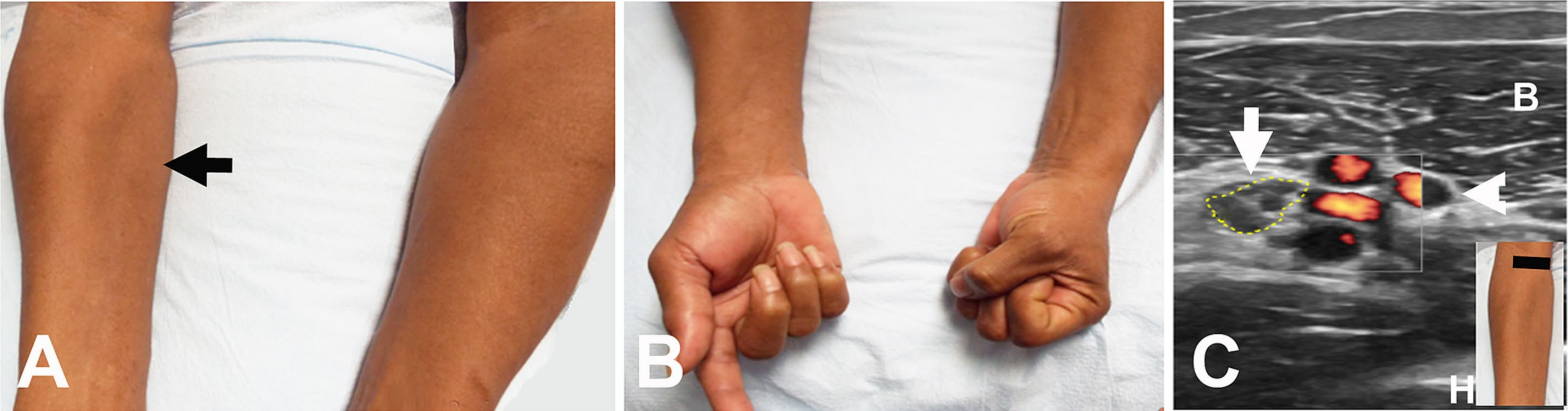
Acute onset of symptoms following intravenous access for a transjugular procedure. **(A)** Wasting of the pronator teres on the right (arrow). **(B)** Weakness of the flexor pollicis longus and flexor digitorum profundus on the right. **(C)** Short axis view at the distal right upper arm showing enlarged median nerve (encircled by yellow dots) adjacent to the brachial artery and vein (arrowhead). The black line in the insert indicates the level of ultrasound. B, biceps muscle; H, humerus.

**Figure 4:**
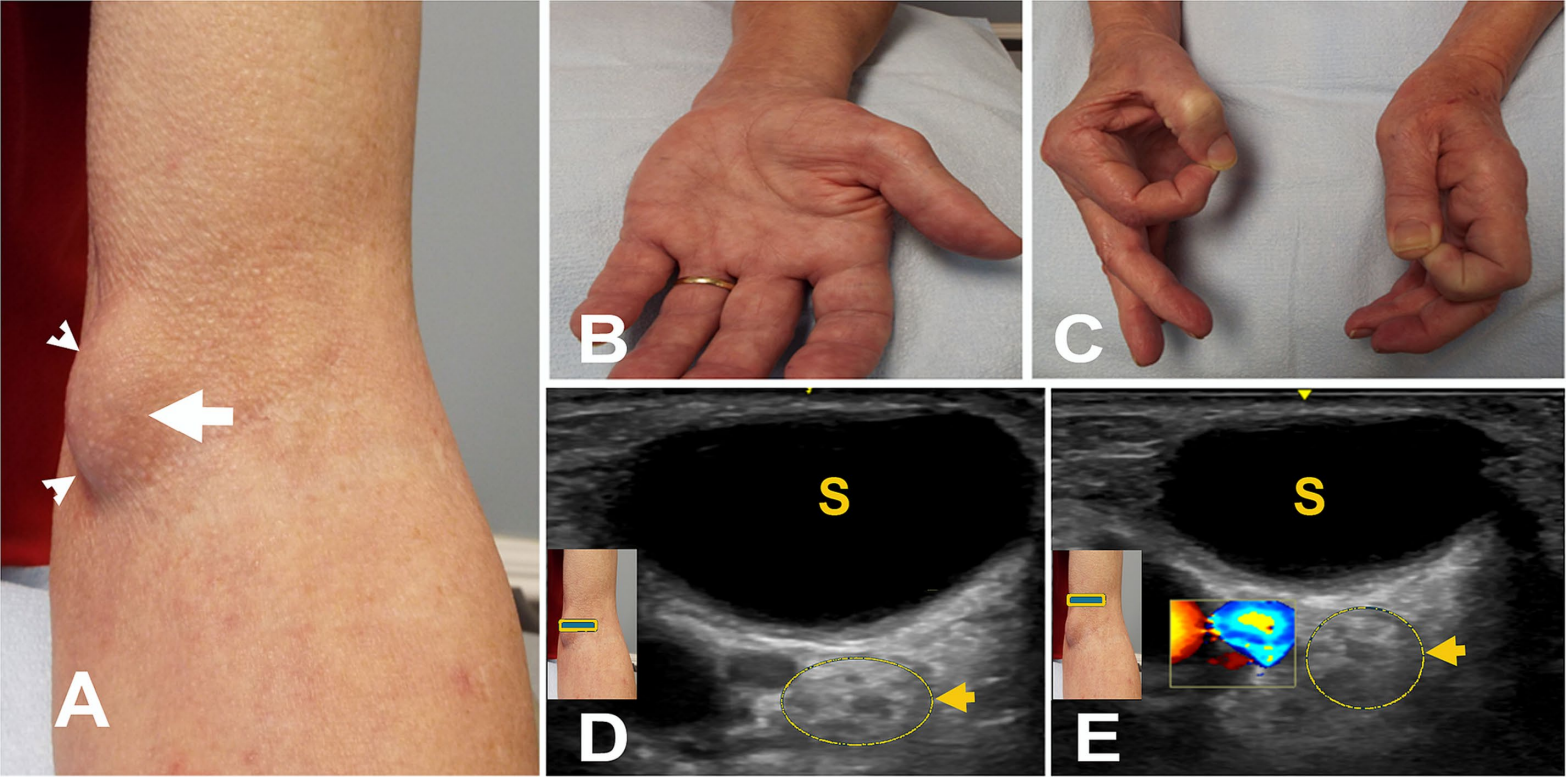
**(A)** Mass after brachial artery cannulation (arrowheads). **(B)** Atrophy of left thenar muscles. **(C)** Positive O sign on the left. **(D)** Short axis view at the elbow with color Doppler over the mass (square box) showing no blood flow. The arrow points to the hyperechoic enlarged median nerve with a few fascicles. **(E)** Short axis view with color Doppler (square box) 5 mm proximal to “A” showing the brachial artery with hyperechoic enlarged median nerve with a few fascicles (arrow). S, seroma.

The most common etiology for all 62 patients was iatrogenic (30 [48.4%]), followed by non-iatrogenic trauma (20 [32.2%]) (Table 2). In Zone 1, iatrogenic injury was the most frequent etiology (25 [65.8%] patients), primarily due to shoulder surgery (7 patients [28.0%]) and brachial artery puncture (5 patients [20.0%]) (Figures 3A–C, 4A–E). The etiology of PMN neuropathies was excessive or undue exertion in 3 (50.0%) patients in Zone 2, PaTS in 5 patients (71.4%) in Zone 3, and non-iatrogenic trauma in 9 patients (81.8%) in Zone 4 (3 [33.3%] patients with a laceration injury and 3 [33.5%] patients with a penetrating injury).

### 3.3 Electrodiagnostic studies

The findings of the EDX studies are summarized in Table 3. The following EDX findings were observed: prolonged distal motor latency (29 [46.8%]), decreased motor nerve conduction velocity in the forearm (22 [35.5%]), low amplitude or absent compound muscle action potentials (50 [80.6%]), and abnormal or absent sensory nerve action potentials (50 [80.6%]). In Zone 1, 36 (94.7%) patients had either abnormal or no SNAP, 33 (86.8%) had either low amplitude or no CMAP, and 17 (44.7%) had prolonged distal motor latency. Low amplitude or no CMAPs were detected in 5 (83.3%) patients in Zone 2, a prolonged distal motor latency was observed in 4 (57.1%) patients in Zone 3, and abnormal/no SNAPs were identified in 11 (100%) patients in Zone 4. In Zone 1, the needle EMG for the FPL and PT was abnormal in all 38 (100%) patients, and the APB was abnormal in 37 (97.4%). A needle EMG abnormality was observed in 6 patients in Zone 2 (APB and FPL), 7 patients in Zone 3 (FPL), and 11 patients in Zone 4 (APB).

### 3.4 Ultrasound studies

**Figure 5:**
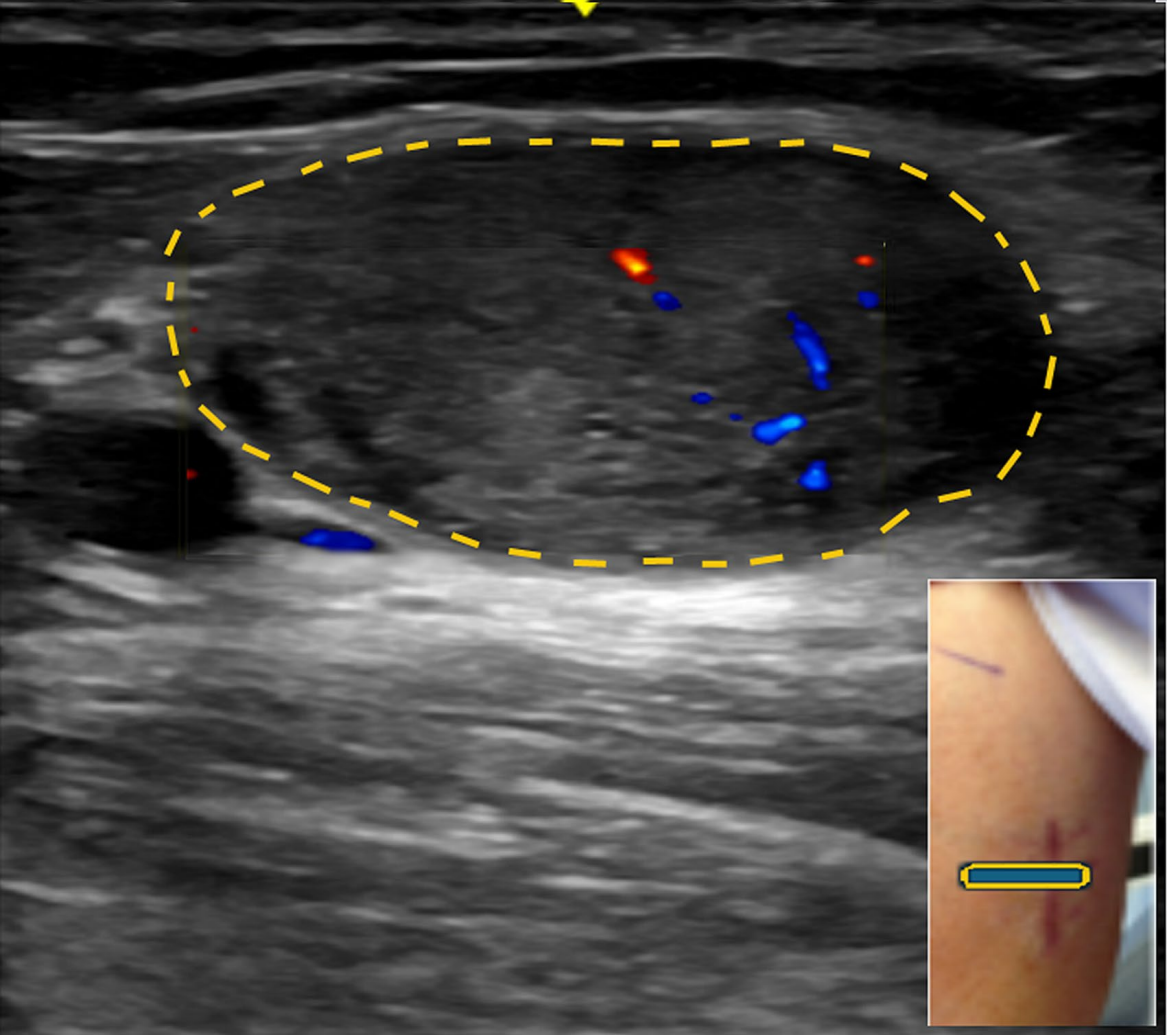
Short axis view with color Doppler at the mid-upper arm showing a schwannoma in the median nerve.

**Figure 6:**
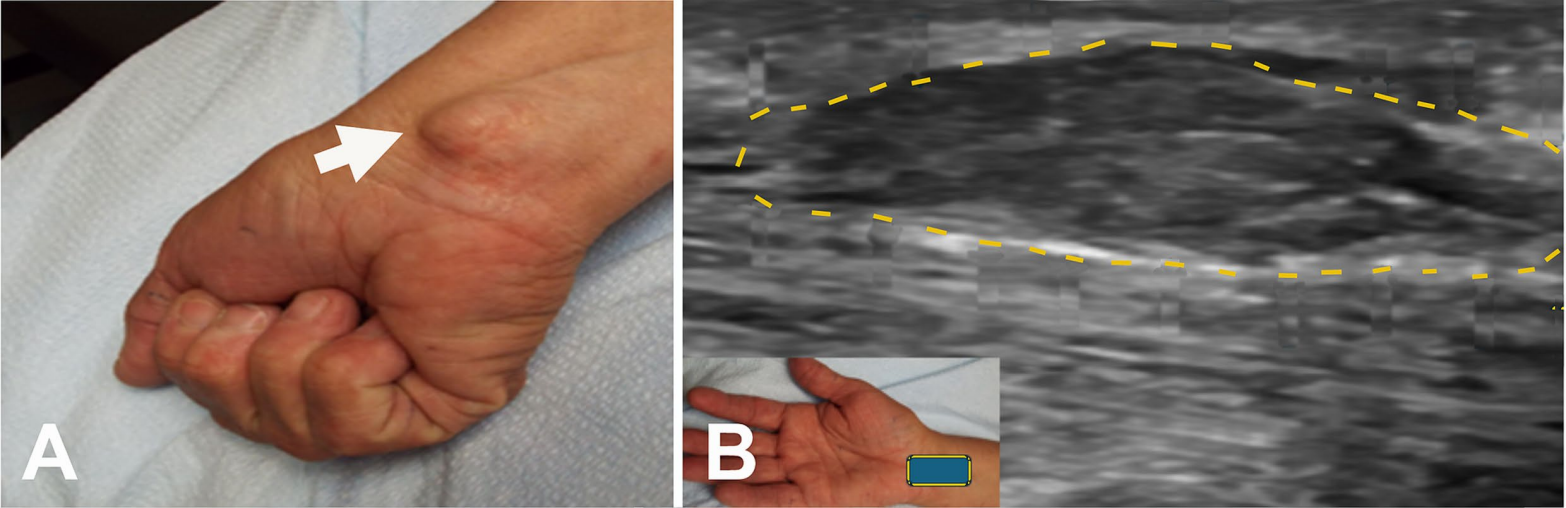
**(A)** Swelling of the right anterior forearm, representing the neuroma (arrow). **(B)** Long axis view at the distal forearm showing median nerve with neuroma in continuity encircled by yellow dots.

Fifty-two (83.9%) patients underwent US studies (Table 3; Figure 5). A total of 23 (42.3%) patients showed an increased CSA of the median nerve. The median nerve was hyperechoic in 16 (30.8%) patients and hypoechoic in 6 (11.5%) patients. A neuroma was observed in 9 (17.3%) patients (with continuity in 7 [77.8%] patients and fascicular discontinuity in 2 [22.2%] patients) (Figures 6A,B). The US studies were normal in 10 (19.2%) patients. A hyperechoic (14 [46.7%]) median nerve with an increased CSA (12 [40.0%]) was commonly observed in Zone 1. Seven (63.6%) patients in Zone 4 had evidence of a neuroma.

## 4 Discussion

The differential diagnoses of PMN neuropathy include CTS, thoracic outlet syndrome (TOS), other brachial plexopathies, and cervical radiculopathy (1). EDX studies are the primary modality for confirming anatomical localization of the site of PMN and determining the underlying pathology and its severity (16). Slowing of motor and sensory conduction, along with alterations in the morphology and amplitude of CMAP and SNAP are metrics used in this assessment (6, 20). EDX studies are useful in differentiating PMN neuropathy from CTS, although they are usually inconclusive or negative in clinically diagnosed PT syndrome (1, 16, 19). The needle EMG should include muscles innervated through the entire length of the median nerve so that the topography of abnormal muscles can be mapped (14). Denervation/reinnervation changes are identified in the median nerve-innervated muscles at and distal to the lesion site (10).

When the lesion is in Zone 1 (involving fascicles in the brachial plexus that form the median nerve and the median nerve itself up to the branch to the PT), precise localization by EDX studies can be challenging. The addition of US can provide further insight. High-resolution US provides high soft-tissue and spatial resolution, cost-effectiveness, safety, ready accessibility, easy portability, and real-time and dynamic nerve imaging in a timely manner (2, 5, 8, 12). US complements EDX studies in providing clues to localizing the lesion and insight into the underlying pathology in patients with PMN neuropathy. There are several scenarios where US is invaluable for the localization of median nerve neuropathy. In Zone 1, nerve conduction studies (NCS) and needle EMG do not always provide adequate findings for localization. Denervation in the PT (the most proximal muscle innervated by the median nerve) makes it difficult to differentiate between lesions at the site of the branch to the PT and more proximal lesions in the upper arm and axilla. US can locate the lesion in most circumstances. Similarly in Zone 4 lesions (in the forearm distal to the origin of the AIN) with denervation of the APB, differentiation from entrapment at the carpal tunnel is difficult. When motor conduction is slow proximal to the wrist, it is not necessarily a conclusive finding as retrograde slowing of motor conduction can occur in longstanding entrapment at the carpal tunnel (25). Precise localization may also be challenging when only the sensory fascicles are affected. While an inching study across the carpal tunnel can be helpful, detecting lesions in the distal forearm may be problematic. US can be useful in detecting such lesions in the forearm. When no CMAPs are detected over the APB and second lumbrical and no SNAPs are recordable over the digits, US is crucial for localization.

US is also useful in identifying the underlying pathology in two specific situations: (1) detection of lesions such as a schwannoma or other tumors/cysts compressing the nerve and (2) distinction between neurotmesis and axonotmesis in cases of nerve injuries. After Wallerian degeneration occurs distal to the site of injury, no conduction can be documented distal to the injury (unlike in a conduction block). While documentation of neurotmesis dictates quick surgical repair, in cases of axonotmesis, one could wait to see if reinnervation occurs within the expected period, which also depends on the length of the injured segment. EMG cannot make the distinction between total axonotmesis and neurotmesis, while US is able to in the majority of circumstances.

Fascicular lesions are particularly important in cases of inflammatory changes. A fascicular somatotopia can be seen early on in the nerve, which may explain the pattern of muscle paresis and sensory disturbance of more proximally located lesions (26). The fascicular anatomy of the median nerve has been well-described, and precise localization by nerve conduction and extensive needle EMG may fail. In PaTS, fascicular involvement proximal to the location suggested by the clinical picture has been reported (26), and our patients did not undergo MR neurography to localize the lesion.

The majority of the studies of PMN neuropathy have focused on entrapment/injury at the elbow and proximal forearm (13, 14, 27). In the study by Sos et al., 53 patients (55 cases, including 2 bilateral) with median nerve entrapment syndrome in the elbow and proximal forearm underwent surgical release of the median nerve and AIN around the elbow and/or proximal forearm. The most common compressed structure was isolated proximal PT insertion (14). The mean age at diagnosis was 56 years with a male-to-female ratio of 1:1 (29 male patients and 26 female patients). The symptom onset was gradual in 32 (58%) patients and acute in 23 (42%) patients. EMG studies demonstrated reduced motor conduction velocity in 38 (56%) patients, conduction block in 5 (9%) patients, and median nerve territory EMG abnormalities in 47 (85%) patients. Three patients had no EMG abnormalities. In Olehnik and colleagues’ study of 36 patients (39 limbs) who underwent surgical decompression of the median nerve in the proximal forearm, the site of compression was the FDS in 22 patients, PT in 13 patients, and both in 4 patients (13). The average age was 39 years, and 29 (80%) were female. Of the 37 limbs that underwent motor and sensory nerve conduction tests, 12 showed an abnormal nerve conduction velocity from the elbow to the wrist. Motor and sensory conduction abnormalities were detected in 12 and 2 limbs, respectively. In Gross and Jones’ study of 17 patients with PMN neuropathy who underwent EMG, the cause of neuropathy was trauma in 5, overuse of the PT in 3 patients, post-infection in 2 patients, secondary to a congenital lesion in 1, and undetermined in 6 (27). The main branch of the median nerve at or proximal to the PT was identified in 14 patients and the AIN in 3. The EMG demonstrated that median nerve compression by the PT caused denervation of the PT and distal muscles. EMG was unable to differentiate a median nerve lesion at the PT from a more proximal lesion (27). Our study concurs with the study by Sos et al. with respect to a similar mean age of patients (56.7 years). However, our study features a higher percentage of male patients (58.1%), acute symptom onset (95.2%), and iatrogenic etiology (48.4%). Our study was also the only one that presented US findings that revealed 42.3% of patients with an increased CSA of the median nerve and 30.2% of patients with a hyperechoic median nerve. All patients in our study underwent EDX studies, which showed low amplitude or no CMAP in 82.2% of the patients and abnormal/no SNAP in 79.0% of the patients.

Traumatic neuropathies were referred for EMG at our neurodiagnostic center after several weeks to months, and, therefore, we could classify them into only conduction block or axonal injury. US was helpful in showing neuroma in continuity versus neurotmesis. However, we were not successful in detecting hourglass fascicular lesions with US in our study.

### 4.1 Strengths and limitations

The strength of the current study is the large number of patients with PMN neuropathy, confirmed by EDX studies. A large percentage of patients in this series also underwent US studies. By determining the etiology of all patients with PMN neuropathy neuropathy, the most effective treatment course may be pursued. Limitations of the present study include its retrospective nature and lack of follow-up after the EDX and US studies as the majority of patients were evaluated only once.

## 5 Conclusion

Neurologists should be aware of the four localization zones of the PMN neuropathy with their distinctive etiologies and utilize additional investigations to confirm the diagnosis and formulate appropriate management. EDX and US studies are important techniques that complement each other in determining the specific localization zone for the PMN neuropathy and providing valuable clues to the underlying pathology in patients with PMN neuropathy. The classification into the four localization zones should be based on the final localization. The suspected lesion may initially be established by the clinical findings, with subsequent details offered by the EDX studies, and final exact localization with the assistance of the US studies. This tiered approach emphasizes the importance of the different modalities. Further studies are warranted to determine the contribution of inflammatory changes.

## Data Availability

The original contributions presented in the study are included in the article/supplementary material, further inquiries can be directed to the corresponding author.
